# RBX1/ROC1-SCF E3 ubiquitin ligase is required for mouse embryogenesis and cancer cell survival

**DOI:** 10.1186/1747-1028-4-16

**Published:** 2009-08-06

**Authors:** Lijun Jia, Yi Sun

**Affiliations:** 1 Division of Radiation and Cancer Biology, Department of Radiation Oncology, University of Michigan Comprehensive Cancer Center, 4424B Medical Science-I, 1301 Catherine Street, Ann Arbor, MI 48109, USA

## Abstract

RBX1 (also known as ROC1) is a RING subunit of SCF (Skp1, Cullins, F-box proteins) E3 ubiquitin ligases, required for SCF to direct a timely degradation of diverse substrates, thereby regulating numerous cellular processes under both physiological and pathological conditions. Previous studies have shown that RBX1 is essential for growth in yeast, *Caenorhabditis elegans *and *Drosophila*. The role of RBX1 in mouse development and in regulation of cancer cell survival was unknown. Our recent work demonstrated that RBX1 is an essential gene for mouse embryogenesis, and targeted disruption of RBX1 causes embryonic lethality at E7.5 due to hypoproliferation as a result of p27 accumulation. We also showed that RBX1 is overexpressed in a number of human cancers, and siRNA silencing of RBX1 caused cancer cell death as a result of sequential induction of G2-M arrest, senescence and apoptosis. These findings reveal a physiological role of RBX1 during mouse development and a pathological role for the survival of human cancer cells. Differential outcomes between normal (growth arrest) and cancer cells (cell death) upon RBX1 disruption/silencing suggest RBX1 as a valid anticancer target.

Comments on:

Tan M, Davis SW, Saunders TL, Zhu Y, Sun Y. RBX1/ROC1 disruption results in early embryonic lethality due to proliferation failure, partially rescued by simultaneous loss of p27. Proc Natl Acad Sci USA. 2009; 106:6203–6208

Jia L, Soengas MS, Sun Y. ROC1/RBX1 E3 ubiquitin ligase silencing suppresses tumor cell growth via sequential induction of G2-M arrest, apoptosis, and senescence. Cancer Res. 2009; 69:4974–82

## Introduction

**Figure 1:**
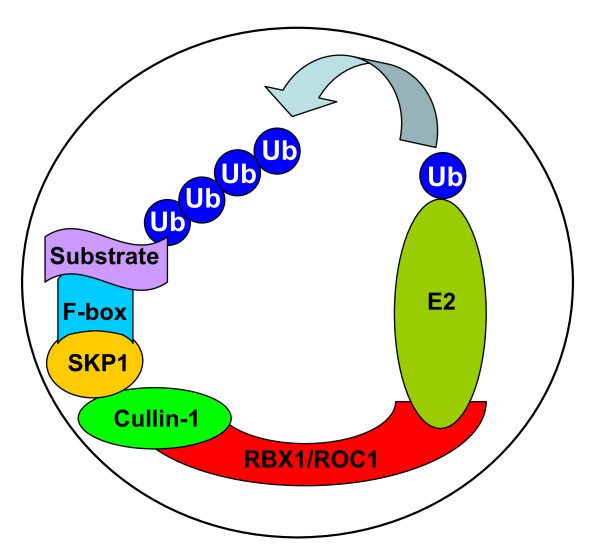
**Substrate ubiquitination by RBX1-SCF E3 ubiquitin ligase**: Cullin-1 at its N-terminus binds to Skp1 and an F-box protein, which recognizes protein substrates, and at its C-terminus binds to RBX1. RBX1, on the other hand, binds to Cullin-1 using its N-terminus and an E2 ubiquitin conjugating enzyme using its C-terminal RING domain. Together, RBX1-cullin-1 catalyzes the ubiquitin transfer from E2 to protein substrates.

SCF (Skp1, Cullins, F-box proteins) E3 ubiquitin ligases, consist of Skp1, Cullins, F-box proteins, and the RING domain containing protein RBX1/ROC1 or its family member RBX2/ROC2/SAG. By promoting degradation of many short-lived proteins, including cell cycle regulators, transcription factors and signal transducers, RBX1-SCF E3 ligases regulate many biological processes. As an essential subunit of the SCF E3 ligase complex, RBX1, is evolutionarily conserved from plants to mammals with multiple family members in each species [1]. Human RBX1 gene consists of five exons and four introns and encodes a 108 amino acids-containing protein with a RING-H2 finger domain (C3H2C3) at the C-terminus, which is required for zinc ion binding and ubiquitin ligation. Human RBX1 is ubiquitously expressed in human tissues with the highest expression in heart, skeleton muscle, kidney and placenta [2]. Structurally, RBX1 binds to the C-terminus of cullin-1 via its N-terminus and to an E2 ubiquitin conjugating enzyme via its C-terminal RING domain. RBX1/Cullin-1 complex catalyzes the ubiquitin transfer from E2 to the substrates which are recognized by different F-box proteins, linked to the N-terminus of cullin-1 via an adaptor protein, Skp1 [3] (Fig 1).

RBX1 binds to all seven cullin family members, including cullin-1, -2, -3, -4A, -4B, -5 and -7 [4]. By binding to and interaction with different cullins and cullin-associated proteins, RBX1, as an active RING component of the largest family of E3 ubiquitin ligases, plays an essential role in regulation of diverse biological processes by promoting the degradation of different protein substrates. Table 1 lists some known substrates targeted by different RBX1-cullins complexes. Over 350 potential RING-cullin substrates were recently identified by a global protein stability profiling analysis [5]. Further characterization and validation of these substrates will broaden our understanding of how RBX1-cullin-based E3 ligases regulate cellular processes under physiological and pathological conditions.

**Table 1:** RBX1-Cullins E3 ubiquitin ligases and their substrates in mammals

**Name**	**Substrates**	**References**
RBX1/Cullin-1/SKP1/F-Box proteins	e.g. p21, p27, p57, Cyclins A/D/E, E2F1, Cdc25A/B, PDCC4, FOXO1, Myc, p53, c-Jun, Notch 1/4, IκB, β-Catenin, Orc1, and many more. For near complete list, see cited references	[14,25,26]

RBX1/Cullin-2/Elongin BC/VHL	e.g. HIF-α, TEL-JAK2	[27,28]

RBX1/Cullin-3/BTB-domain proteins	e.g. MEI-1, Dishevelled (Dsh), Nrf2, RhoBTB2, topoisomeraseI-DNA complex, and caspase 8	[29-36]

RBX1/Cullin-4A/DDB1	e.g. p53, TSC2, Cdt1, c-Jun and Merlin	[37-45]

RBX1/Cullin-5/elongin BC/BC-box proteins/SOCS	e.g. Disabled-1 (Dab1)	[46]

RBX1/Cullin-7/SKP1/Fbw8	e.g. Insulin receptor substrate 1 (IRS-1)	[47]

## Discussion

### RBX1 in development

RBX1-cullin complexes control the proteolysis of numerous substrates related to cell cycle progression, cell growth and cell death, suggesting that RBX1 may play an important role in development. Indeed, RBX1 is an essential gene in a number of species. In *yeast*, deletion of *Hrt1*, the yeast homologue of RBX1, via genetic manipulation, causes yeast death, which can be rescued by human RBX1 or RBX2/SAG [6-8]. In *Caenorhabditis elegans*, RBX1 is also crucial for cell cycle progression and chromosome metabolism, as evidenced by severe defects in meiosis, mitotic chromosomal condensation and segregation, and cytokinesis upon siRNA knockdown [9]. In *Drosophila*, ROC1a, the drosophila homologue of RBX1 is required for cell proliferation and embryo development, and deletion of ROC1a results in animal death [10]. In mouse, the level of RBX1 mRNA was regulated during embryonic development with the strongest expression at embryonic day 7 (E7), followed by a progressive decrease [11]. However, the physiological role of RBX1 in mouse development has not been previously characterized.

**Figure 2:**
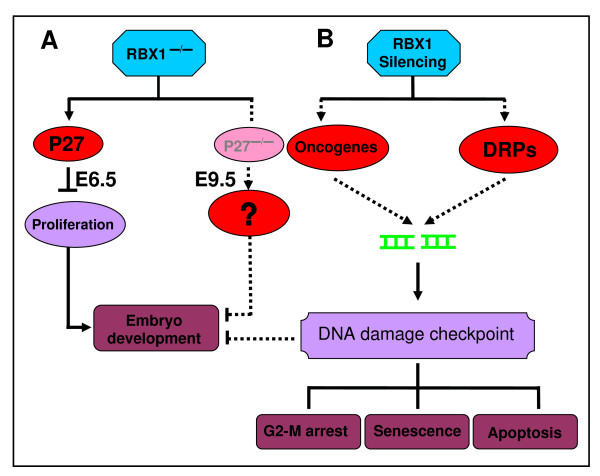
**A working model for RBX1 targeting**. **(A) In mouse embryos**. RBX1 disruption in mouse induces early embryonic lethality due to reduced proliferation as a result of p27 accumulation. Simultaneous deletion of p27 restores cell proliferation and causes a partial rescue of embryonic death by extending the embryo's life from E6.5 to E9.5. It is unclear, at the present time, if abnormal DNA damage response is involved in later stage death of RBX1/p27 double null embryos. **(B) In human cancer cells**: RBX1 silencing triggers DNA damage response and checkpoint controls via modulating the levels of oncogenes or DNA replication proteins (DRPs), leading to activation of multiple cell killing pathways, including G2-M arrest, senescence and apoptosis.

Most recently, we characterized the *in vivo *physiological function of RBX1 during mouse development using a conventional knockout study [12]. We found that homozygous disruption of mouse *RBX1 *via a gene trap strategy causes embryonic lethality at E7.5 as a result of reduced proliferation, but not enhanced apoptosis. Mechanistic studies revealed that *RBX1 *disruption induces significant accumulation of p27, a cyclin dependent kinase inhibitor, normally not expressed in early embryos. The p27 accumulation was further observed in mouse embryonic fibroblasts (MEF) or mouse embryonic stem cells (ESC) with *RBX1 *heterozygous background. In *RBX1*^+/- ^MEF cells, p27 accumulation is associated with growth retardation and G1 arrest. Causal involvement of p27 accumulation in early death of *RBX1*-deficient embryos was clearly demonstrated by a rescue experiment in which simultaneous loss of p27 extends the life span of *RBX1*-deficient embryos from E6.5 to E9.5 [12]. Our study demonstrates that the *in vivo *physiological function of RBX1 is to ensure cell proliferation by preventing p27 accumulation during the early stage of embryonic development (Fig 2A). The fact that p27 loss cannot completely rescue *RBX1*-deficient embryos indicates that accumulation of other RBX1 substrates upon *RBX1 *disruption is detrimental to embryonic development beyond E9.5 (Fig 2A). A future challenge will be to define these physiologically relevant substrates to broaden our understanding of the *in vivo *physiological function of RBX1 in the later stages of mouse embryogenesis.

Functional characterization using various model systems from yeast to mouse clearly demonstrated that *RBX1 *is an essential gene for growth and development. Interestingly, some similarity and difference exist between the species with more than one family member of RBX1. For example, death phenotype induced by disruption of *ROC1a *in *Drosophila *or of *RBX1 *in mouse in the presence of their family member *ROC2 *or *RBX2 *[10,12,13] clearly indicated that these two RBX1 family members are not functionally redundant and are likely to target different sets of substrates during embryonic development. On the other hand, the mechanisms responsible for reduced proliferation as a result of *ROC1a*/*RBX1 *disruption seem different between *Drosophila *and mouse. In *Drosophila*, disruption of *ROC1a*, causes lethality due to proliferation failure as a result of accumulation of Ci (a *Drosophila *ortholog of mouse Gli2), a transcription factor that regulates Hedgehog signaling [10]. Whereas in mouse, disruption of *RBX1 *does not affect the levels of Gli2, but causes p27 accumulation to suppress proliferation [12]. Furthermore, although *RBX1-p27 *double null embryos at E9.5 are smaller than wild type littermates, no enhanced apoptosis was detected using the TUNEL assay (unpublished data). Thus, during mouse embryogenesis, *RBX1 *disruption appears not to induce apoptosis. These observations in normal tissues are strikely different from those seen in human cancer cells (see below) in which RBX1 silencing induces significant levels of apoptosis and senescence.

### RBX1 in human cancer cell survival

RBX1-SCF E3 ubiquitin ligases regulate numerous cellular processes. It is not surprising that their dysfunction is associated with a variety of diseases including cancer [14]. For example, an oncogenic F-box protein Skp2, which promotes p27 degradation, is overexpressed in a number of human cancers [15], whereas a tumor suppressive F-box protein FBW7, which promotes the degradation of several proto-oncogenes, including c-Jun, c-Myc, cyclin E and mTOR undergoes numerous cancer-associated mutations [16]. To define potential roles of RBX1 in human cancers, we recently measured expression of RBX1 in human primary cancer tissues and in cancer cell lines with different tissue origins. We found that RBX1 is overexpressed in a number of human primary cancer tissues, including carcinoma of lung, liver, breast, colon, and ovary, and in many cancer cell lines [17]. We then determined potential biological consequences of reducing RBX1 levels via siRNA silencing. Significantly, RBX1 knockdown inhibited the growth of several human cancer cell lines by sequential induction of G2-M arrest, senescence and apoptosis. Further characterization revealed that G2-M arrest is associated with accumulation of 14-3-3σ and down-regulation of cyclin B1 and Cdc2, whereas apoptosis is associated with modest accumulation of PUMA and significant reduction of Bcl-2, Mcl-1, and survivin. Interestingly, senescence is p53/p21- and p16/pRB-independent [17]. Recently a shRNA library-based functional genomic screen also identified *RBX1 *as a growth essential gene in a number of human cancer cell lines, although no characterization was further pursued [18].

Mechanistic studies revealed that RBX1 silencing triggers DNA damage response at the early stage, as demonstrated by induced phosphoralytion of H2AX, Chk1 and Chk2 [17] (and unpublished data), which eventually leads to G2-M arrest, followed by apoptosis and senescence. We hypothesize that either or both sets of RBX1 substrates, which start to accumulate upon RBX1 silencing, are likely involved in the process, leading to phenotypic changes. The first set includes oncogenes (e.g. c-Myc, c-Jun, cyclin E/D), since oncogene activation triggers DNA damage response to induce senescence and apoptosis under certain circumstances [19,20]. The second set of RBX1-cullin substrates could be DNA replication proteins, such as Orc-1 and Cdt-1, since the accumulation of DNA replication proteins (e.g. Cdt-1) induces DNA rereplication stress and triggers DNA damage [21] (Fig 2B). Our laboratory is currently testing the hypothesis to further elucidate the mechanism(s) by which RBX1 silencing induces cancer cell killing via induction of G2-M arrest, senescence and apoptosis.

It is rather clear that the mechanism responsible for early embryonic lethality upon RBX1 disruption is quite different from that responsible for cancer cell killing upon RBX1 silencing, although it is not a typically paired comparison. Nevertheless, in mouse, RBX1 knockout leads to p27 accumulation, reduced proliferation and prolonged G1 arrest, whereas in human cancer cells, p27 accumulation and G1 arrest were not observed upon RBX1 silencing [17]. Future studies should be directed to determine if the altered DNA damage response commonly seen in cancer cells after RBX1 silencing can also be observed in RBX1-p27 double null embryos and if so, its contribution to the embryonic lethality in the later stages of development (Fig 2, crosstalk between panel A and B).

## Conclusion

The findings from our laboratory demonstrated that RBX1 is an essential gene not only for mouse development but also for human cancer cell survival. The fact that RBX1 is overexpressed in a number of human cancers suggests that abnormal regulation of RBX1 is involved either in human carcinogenesis or in the maintenance of the cancer cell phenotype. Differential response to RBX1 disruption/silencing between normal tissues (reduced proliferation, but no induction of apoptosis during mouse embryogenesis) and cancer cells (enhanced cell killing) may provide a reasonable therapeutic window for cancer cell-specific killing via RBX1 targeting. Thus, future development of siRNA-based therapy by RBX1 silencing or small molecule inhibitors against RBX1 E3 ubiquitin ligases may hold great promise for the treatment of human cancer [22-24].
